# Characterization of Plasmid-Mediated Quinolone Resistance and Serogroup Distributions of Uropathogenic *Escherichia coli* among Iranian Kidney Transplant Patients

**DOI:** 10.1155/2020/2850183

**Published:** 2020-10-27

**Authors:** Amin Sadeghi, Mehrdad Halaji, Amirhossein Fayyazi, Seyed Asghar Havaei

**Affiliations:** Department of Microbiology, School of Medicine, Isfahan University of Medical Sciences, Isfahan, Iran

## Abstract

**Introduction:**

Urinary tract infection (UTI) is one of the most frequent infections in kidney transplant patients (KTPs). This infection is mainly caused by uropathogenic *Escherichia coli* (UPEC). Plasmid-mediated quinolone resistance (PMQR) was also increasingly identified in UPEC. This study proposed to investigate the frequency of quinolone-resistance plasmid genes and the O-antigen serogroup among UPEC isolated from KTPs and non-KTP with UTI.

**Methods:**

Totally, 114 UPEC isolates from 49 KTPs and 65 non-KTPs patients diagnosed with an UPEC-associated UTI were obtained from June 2019 to December 2019 at three laboratory centers in Isfahan, Iran. The isolates were confirmed through phenotypic and genotypic methods. Moreover, the antimicrobial susceptibility test to nalidixic acid, ciprofloxacin, norfloxacin, and ofloxacin was performed using a disk diffusion method. The presence of the *qnr* gene as well as the serogroup distribution was identified using the PCR method.

**Result:**

According to data, the distribution of *O1*, *O2*, *O4*, *O16*, and *O25* serogroups were 3.5%, 2.6, 3.5, 3.5, and 20.2%, respectively. Antibiotic susceptibility pattern revealed that the highest and lowest resistance rates were to nalidixic acid (69.3%) and norfloxacin (43.9%), respectively. Also, the frequency of *qnrS* and *qnrB* genes were 33.3% and 15.8%, respectively, while none of the isolates was found to be positive for the *qnrA* gene. There was no significant association between the presence of *qnr* genes and higher antibiotic resistance.

**Conclusion:**

This study recognized that the *qnrS* gene, *O25* serotype, and resistance to nalidixic acid had the highest frequencies in UPEC strains isolated from UTI patients.

## 1. Introduction

Urinary tract infection (UTI) has been recognized as the second-ranking infectious disease worldwide that is, and UTIs are known as a serious global concern in public health care systems [1]. Moreover, UTIs are one of the most frequent infections in kidney transplant patients (KTPs) [2]. The effects of UTI on kidney transplantation (KTx) have been well reported in previous studies [3]. UTI occurs in 60% of KTPs patients during the first year posttransplant [4]. Almost 70% of these UTIs are caused by gram-negative bacteria [3]. *Escherichia coli* (*E. coli*) strains, in particular, uropathogenic *E. coli* (UPEC) pathotype, are known as the most frequent gram-negative bacteria causing UTIs after KTx [1, 5]. On the other hand, antibiotic therapy of UTI has become problematic due to misuse of antibiotics which can in turn give rise to the emergence of resistant strains [6]. Quinolones are a group of artificial antimicrobial agents with a broad antibacterial activity, frequently used as a treatment in patients with UTI. These groups have been divided into four generations based on their antimicrobial activity; nalidixic acid, ciprofloxacin, and levofloxacin that are members of the first, second, and third generations, respectively [6, 7]. Quinolones, in particular, ciprofloxacin, is one of the agents frequently utilized for treatment of UTIs. Moreover, it is considered as an effective treatment in prevention of UTI in KTPs [8]. DNA gyrase (topoisomerase II) and topoisomerase IV are named as the primary and secondary targets for quinolones [6]. The extent of quinolone-resistant genes in gram-negative bacteria like UPEC is a big concern for KTPs [1]. Quinolone resistance (*qnr*) is caused by several mechanisms which plasmid-mediated quinolone resistance (PMQR) is one of the most important of them. Moreover, PMQR is approved by the *qnr* genes. The qnr genes contain *qnrA*, *qnrB*, *qnrC*, and *qnrS* [6, 9]. *Qnr* proteins are protecting target enzymes DNA gyrase and topoisomerase IV of quinolone inhibition [7]. Further, PMQR provides only a low level of quinolone resistance; however, PMQR genes may facilitate the selection of higher-level resistance in the presence of quinolones and lead to treatment breakdown [7]. The *E. coli* strains are commonly classified on the basis of serological typing of their H (flagellar), O (lipopolysaccharide), and in some cases, and K (capsular) surface antigens [10]. To date, although more than 174 different serogroups have been reported for *E. coli*, some O-antigen types usually expressed in UPEC clones, including *O1*, *O2*, *O4*, *O6*, *O7*, *O8*, *O16*, *O18*, *O25*, and *O75* [11]. To the best of our knowledge, few studies have investigated quinolone resistance and O-serogroups in UPEC strains among KTPs. Therefore, this study was designed to determine the prevalence of quinolone resistance and O-serogroups (*O1, O2, O4, O16, and O25*) in UPEC strains isolated from Iranian KTPs and non-KTPs with UTIs.

## 2. Material and Method

### 2.1. Study Design and Bacterial Isolation

This cross-sectional study conducted from June 2019 to December 2019, in three laboratory centers and two nephrology private clinics in Isfahan, Iran. 65 nonrepetitive UPEC isolates obtained from non-KTP with UTIs, and 49 nonrepetitive UPEC isolates obtained from KTPs with UTIs. This study was evaluated and approved by the Ethics Committee of Isfahan University of Medical Sciences (IR.MUI.MED.REC.1398.196). The UPEC isolates were confirmed as *E. coli* by gram staining and the standard biochemical tests in previously described [12]. The confirmed isolates were stored at -80°C in brain heart infusion broth containing 20% glycerol.

### 2.2. Quinolone Susceptibility Testing

The antibiotic susceptibility pattern was determined based on the disk diffusion method on Mueller–Hinton agar (HiMedia Co., India) according to the Clinical and Laboratory Standards Institute (CLSI) [13] recommendation for nalidixic acid, ciprofloxacin, norfloxacin, and ofloxacin (BD BBL™ Sensi-Disc™).

### 2.3. Detection of *qnr* Encoding Genes

Genomic DNA was extracted from fresh colonies as described previously [14]. PCR was performed to detect the presence of *qnrA*, *qnrB*, and *qnrS* genes using the specific primers [15]. The conditions for PCR amplification were initial denaturation at 94°C for 5 minutes, followed by 30 cycles of denaturation at 94°C for 30 seconds, primer annealing at 55°C for *qnrA* and *qnrB* and 58°C for *qnrS* for 30 seconds, extension at 72°C for 30 seconds, and a final extension at 72°C for 5 minutes. Amplification products were analyzed using 1.5% agarose gel with KBC power load dye (CinnaGen Co. Iran). Positive results were confirmed by direct sequencing of the PCR products.

### 2.4. Characterization of the Lipopolysaccharide O Antigens

PCR was performed to detect the presence of *O1, O2, O4, O16*, and *O25* genes using the specific primers [11]. The conditions for PCR amplification were initial denaturation at 94°C for 5 minutes, followed by 30 cycles of denaturation at 94°C for 30 seconds, primer annealing at 55°C for *O1* and 58°C for *O2* and *O25* and 56°C for *O4* and *O16* for 30 seconds, extension at 72°C for 30 seconds, and a final extension at 72°C for 5 minutes. Amplification products were analyzed using 1.5% agarose gel with KBC power load dye (CinnaGen Co. Iran).

The statistical analysis was done using SPSS software version 16.0 (IBM Corp., USA). For this study, Fisher's test and the nonparametric chi-square test were performed, and *P* value <0.05 was considered statistically significant.

## 3. Results

During the period of study, in total, 114 confirmed UPEC was isolated, including 43% (49/114) KTP and 57% (65/114) control group. Furthermore, among KTP, 69.4% (34/49) and 30.6% (15/49) isolates were female and male patients, respectively, while among non-KTP, 69.2% (45/65) and 30.8% (20/65) isolates were obtained from female and male, respectively. The range age of the study group was from 10 to 80 years. The results of the antibacterial susceptibility test revealed that most isolates were resistant to nalidixic acid 69.3% (79/114), while the least resistance was demonstrated against norfloxacin 43.9% (50/114). The total distribution of the antibacterial susceptibility test is shown in Table 1. UPEC strain isolated from KTP was highly resistant to nalidixic acid 75.5% (37/49) while the least resistance was demonstrated against ofloxacin 51% (25/49) (Table 1). The antibiotic susceptibility pattern on the control group revealed that the highest and lowest resistance rates were against nalidixic acid 64.6% (42/65) and norfloxacin 35.4% (23/65), respectively (Table 1). Statically analysis showed that the resistance rate against norfloxacin was significantly higher among KTP than non-KTP (Table 1).

PCR amplification of the three genes (*qnrA, qnrB* and *qnrS)* showed that 33.3% (38/114) and 15.8% (18/114) of the isolates were positive for *qnrS* and *qnrB* genes, respectively, while none of the isolates was found to be positive for the *qnrA* gene (Table 2). In addition, 3.5% (4/114) of the isolates were found positive for both genes. There was no significant relationship between *qnr* genes and higher quinolone resistance in KTP and control group. However, the *qnrS* gene was found to be relatively higher than the *qnrB* gene among quinolone resistance isolates (Table 2). Accordingly, the *qnr* genes are most likely to happen in nalidixic acid resistant rather than fluoroquinolone-resistant isolates. The total distribution of *qnr* genes among quinolone resistance KTP and non-KTP is presented in Tables 3 and 4. According to our finding, the high distribution of serogroups was *O25* (20.2%, 23/114), while the *O2* serogroup (2.6%, 3/114) was the lowest serogroups among UPEC isolates (Table 2). The total distribution of O-serogroups is summarized in Table 2. Overall, the *O25* serogroup (60.9%, 14/23) had the highest distributions of serogroups while *O2*, *O4*, and *O16* genes were not found in KTP (Table 2).

Of the studied O-serogroups, *O1* and *O25* genes were significantly higher among KTP than the control group (Table 2). All serogroups showed the highest resistance to ciprofloxacin, norfloxacin, and ofloxacin in KTP and nalidixic acid and norfloxacin in the control group (Tables 5 and 6). The results showed the significant resistance rate of ciprofloxacin and norfloxacin between the *O25* serogroup in the control group (Table 6). According to future investigation, among 18 *qnrB*-carrying strains, 8, 1, and 1 isolates belonged to the *O25, O1*, and *O4* serogroup, respectively. In addition, among 38 *qnrS*-carrying strains, 8 and 1 isolates belonged to the *O25* and *O4* serogroup, respectively.

## 4. Discussion

UTI is the most common disease among KTPs [16, 17], and the main cause of UTI is UPEC [18]. Furthermore, quinolones are the most common antibiotic used to treat UTI. Nowadays, fluoroquinolone resistance producing UPEC has increased worldwide [19]. In the present study, among all UPEC isolates, the highest and lowest resistance were to nalidixic acid (54.9%) and norfloxacin (43.8%), respectively. Moreover, the antibiotic susceptibility pattern in KTPs and non-KTPs revealed that nalidixic acid had the most antibiotic resistance with 75.5% (37/49) and 64.6% (42/65) rate, respectively. Therefore, it seems that we should be more cautious about using nalidixic acid and other quinolones with the aim of UTI treatment in our region. In a study conducted on KTPs, Siliano et al. (2010) reported a 31.66%, 28.99%, and 28.99% resistance rate of nalidixic acid, norfloxacin, and ciprofloxacin, respectively [20]. Mohammadzadeh et al. (2019) investigated antibiotic susceptibility of UPEC isolates from KTPs. Their results showed that the resistance rate of ciprofloxacin and norfloxacin was 34.78% and 4.34%, respectively [21]. This finding was not in agreement with our finding.

Additionally, in a study conducted on KTPs by Espinar et al. (2015), among ESBL-positive UPEC isolates, 48.5% and 36.4% of isolates were resistant to ciprofloxacin and norfloxacin [22]. Moreover, Kashef Nejad et al. (2017) in a study on UPEC isolated from KTPs reported that 71.92% of ESBL-producing isolates had resistance toward ciprofloxacin [4]. In a meta-analysis study conducted by Moghaddam et al., among the UPEC strain isolated from KTPs, the resistance rate of quinolones agents to nalidixic acid and ciprofloxacin was reported to be 68.4% and 61%, respectively [23]. These results were in consistent with the results of our study. Furthermore, a study carried out by Shenagari et al. (2018) revealed that among 223 UPEC isolates, nalidixic acid (61.9%) and norfloxacin (45.3%) were the highest antibiotic resistance among quinolone agents [24]. Similarly, in southwest of Iran, Farajzadeh Sheikh et al. found that 65.3% of UPEC isolates were resistant to nalidixic acid [25]. The results of these studies performed across Iran were close to our findings. Malekzadegan et al. (2019) showed that the highest resistance rates were to nalidixic acid (71.9%) among 121 UPEC in a tertiary care hospital in the south of Iran [26]. Our studies showed a lower percentage of antibiotic resistance than that of hospital studies. This indicates that inpatients were more exposed to high antibiotic selective pressure or bacterial crosstransmission. This could be due to the reckless and inappropriate use of drugs. Furthermore, several findings suggested that the source of quinolones resistance and spread of resistance genes may be explained by animals-to-human-transmission routes, such as poultry and meat products, which can be in turn due to inappropriate use of antibiotics in the animal industry. Based on the molecular result, 45.6% (52/114) of the isolates contained *qnr* genes. The most prevalent PMQR genes were *qnrS* 33.3% (38/224) and *qnrB* 15.8% (18/114), while the *qnrA* genes were not found in this study. According to the literature review, the *qnrS* gene seems to play a significant role in quinolone resistance than *qnrA* and *qnrB*, which is consistent with our results among both KTPs and non-KTPs. Tarchouna et al. (2015) reported a frequency of 32% for the *qnr* genes that the most frequent of them was *qnrB* (12.5%), followed by 5.3% for *qnrA*, and 3.5% for qnrS [27]. In multicenter study conducted in the west of Iran by Valadbeigi et al. (2020), results indicated that the frequency of *qnrB* and *qnrS* genes was 47.5% and 2.5%, respectively [28]. The reason for this difference might be due to the hospital samples examined in these studies. Abbasi et al. (2018) further found that the prevalence of *qnrB* and *qnrS* genes was 25%, and 36%, respectively, in Tehran [1]. In agreement with our studies, Röderova et al. (2017) and Rezazadeh et al. (2016) asserted that the *qnrA* genes were not found among the isolates [29, 30]. Systematic O-serogrouping of *E. coli* began in the early 1930s, and it became an important tool for the classification of *E. coli* strains in clinical settings [31]. Much evidence of experimental studies is showing a close connection among certain serogroups and certain markers of pathogenicity in pathogens, such as UPEC [32, 33]. Overall, *O1*, *O2*, *O4*, *O16*, and *O25* were the detected O-serogroups among the UPEC isolates in this study. Moreover, in the current study, 33.3% (38/114) of the isolates detected O-Serogroups. *O25*, 20.2% (23/114), was the most commonly detected O-serogroups in UPEC strains of our study. Our results also indicated that there was several serogroups of *E. coli* in KTP patients with UTI. There were statistically significant differences between the presence of *O25* (*P* < 0.05) and *O1* (*P* < 0.01) serogroups in KTPs in comparison with non-KTPs. Consistent with our result, in a study by Lau et al., on 43 UPEC strains isolated, the most prevalent serogroup was reported *O25* with 21 cases (48.84%) [34]. Noie Oskouie et al. (2019) reported that the gene of *O25* (55.8%) was the most commonly detected serogroups among UPEC [35]. In another study carried out by Basha et al. (2019) among KTPs, the frequency of *O2, O4, O16, and O25* was reported to be 7%, 4.2%, 2.8%, and 5.6%, respectively [5]. Furthermore, according to the results of Momtaz et al.'s study (2013), *O25* (26.01%), *O16* (10.56%), *O4* (5.69%), *O1* (2.43%), and *O2* (2.43%) were the most commonly detected serogroups among Iranian hospitalized patients [36]. Shokouhi Mostafavi et al. (2019) showed that *O1* (20%) and *O25* (13.7%) were the major O-serogroups among UPEC isolates in Iran [37]. According to the distribution of quinolone resistance in various O groups in non-KTPs, the isolates belonged to the *O25* group showed the significant resistance rate to ciprofloxacin and norfloxacin than the other O groups. In general, the different distributions of O-serogroups among UPEC isolates can vary depending on the type of infection, region, or even different settings (hospital or community). Based on our results, *qnrS* gene, *O25* serotype, and finally, resistance to nalidixic acid had the highest frequencies in UPEC strains isolated from UTI patients. We also recommend antibiotics taking only in severe conditions since quinolone resistance has been increased in UPEC strains.

## Figures and Tables

**Table 1:** Antimicrobial susceptibility pattern of UPEC isolates among KTP and control group.

Antibiotic	Total	KTP (49) *N* (%)	Control group (65) *N* (%)	*P* value
Nalidixic acid	79	37(75.5)	42 (64.6)	0.21
Ciprofloxacin	52	27 (55.1)	25 (38.5)	0.07
Ofloxacin	57	25 (51)	32 (49.2)	0.85
Norfloxacin	50	27 (55.1)	23 (35.4)	**0.03**

**Table 2:** Distribution of qnr genes and O-serogroups among KTP and control group.

Genes	Total	KTP (49) *N* (%)	Control group (65) *N* (%)	*P* value
*qnrB*	18 (15.8)	6 (12.2)	12 (18.5)	0.36
*qnrS*	38 (33.3)	15 (30.6)	23 (35.4)	0.59
*O1*	4 (3.5)	4 (8.2)	0 (0)	**0.01**
*O2*	3 (2.6)	0 (0)	3 (4.6)	0.12
*O4*	4 (3.5)	0 (0)	4 (6.2)	0.07
*O16*	4 (3.5)	0 (0)	4 (6.2)	0.07
*O25*	23 (20.2)	14 (28.6)	9 (13.8)	**0.05**

**Table 3:** Distribution of *qnr* genes among quinolone resistance in KTP.

Antibiotic	Pattern	*qnrS*-positive no. (%)	*qnrS*-negative no. (%)	*P* value	*qnrB*-positive no. (%)	*qnrB*-negative no. (%)	*P* value
Nalidixic acid	R (*n* = 37)	10 (27)	27 (73)	0.47	4 (10.8)	33 (89.2)	0.59
	S (*n* = 12)	5 (41.7)	7 (58.3)		2 (16.7)	10 (83.3)	
Ciprofloxacin	R (*n* = 27)	9 (33.3)	18 (66.7)	0.64	3 (11.1)	24 (88.9)	0.78
	S (*n* = 22)	6 (27.3)	16 (72.7)		3 (13.6)	19 (86.4)	
Ofloxacin	R (*n* = 25)	9 (36)	16 (64)	0.40	2 (8)	23 (92)	0.35
	S (*n* = 24)	6 (25)	18 (75)		4 (16.7)	20 (83.3)	
Norfloxacin	R (*n* = 27)	9 (33.3)	18 (66.7)	0.64	3 (11.1)	24 (88.9)	0.78
	S (*n* = 22)	6 (27.3)	16 (72.7)		3 (13.6)	19 (86.4)	

Abbreviations: R: resistant; S: susceptible.

**Table 4:** Distribution of *qnr* genes about quinolone resistance in control-group.

Antibiotic	Pattern	*qnrS*-positive no. (%)	*qnrS*-negative no. (%)	*P* value	*qnrB*-positive no. (%)	*qnrB*-negative no. (%)	*P* value
Nalidixic acid	R (*n* = 42)	12 (28.6)	30 (71.4)	0.12	9 (21.4)	33 (78.6)	0.51
	S (*n* = 23)	11 (47.8)	12 (52.2)		3 (13)	20 (87)	
Ciprofloxacin	R (*n* = 25)	8 (32)	17 (68)	0.65	6 (24)	19 (76)	0.51
	S (*n* = 40)	15 (37.5)	25 (62.5)		6 (15)	34 (85)	
Ofloxacin	R (*n* = 32)	11 (34.4)	21 (65.6)	0.86	7 (21.9)	25 (78.1)	0.48
	S (*n* = 33)	12 (36.4)	21 (63.6)		5 (15.2)	28 (84.8)	
Norfloxacin	R (*n* = 23)	7 (30.4)	16 (69.6)	0.53	6 (26.1)	17 (73.9)	0.31
	S (*n* = 42)	16 (38.1)	26 (61.9)		6 (14.3)	36 (85.7)	

Abbreviations: R: resistant; S: susceptible.

**Table 5:** Antimicrobial resistance pattern in UPEC isolates based on serogroups in KTP.

Antibiotic	Pattern	*O1*-positive no. (%)	*O1*-negative no. (%)	*P* value	*O25*-positive no. (%)	*O25*-negative no. (%)	*P* value
Nalidixic acid	R(*n* = 37)	4 (10.8)	33 (89.2)	0.23	9 (24.3)	28 (75.7)	0.28
	S (*n* = 12)	0 (0)	12 (100)		5 (41.7)	7 (58.3)	
Ciprofloxacin	R (*n* = 27)	3 (11.1)	24 (88.9)	0.40	7 (25.9)	20 (74.1)	0.65
	S (*n* = 22)	1 (4.5)	21 (95.5)		7 (31.8)	15 (68.2)	
Ofloxacin	R (*n* = 25)	2 (8)	23 (92)	0.96	7 (28)	18 (72)	0.92
	S (*n* = 24)	2 (8.3)	22 (91.7)		7 (29.2)	17 (70.8)	
Norfloxacin	R (*n* = 27)	3 (11.1)	24 (88.9)	0.40	7 (25.9)	20 (74.1)	0.65
	S (*n* = 22)	1 (4.5)	21 (95.5)		7 (31.8)	15 (68.2)	

Abbreviations: R: resistant; S: susceptible.

**Table 6:** Antimicrobial resistance pattern in UPEC isolates based on serogroups in the control group.

Antibiotic	Pattern	*O2*-positive no. (%)	*O2*-negative no. (%)	*P* value	*O4*-positive no. (%)	*O4*-negative no. (%)	*P* value	*O16*-positive no. (%)	*O16*-negative no. (%)	*P* value	*O25*-positive no. (%)	*O25*-negative no. (%)	*P* value
Nalidixic acid	R(*n* = 42)	2 (4.8)	40 (95.2)	0.93	2 (4.8)	40 (95.2)	0.52	3 (7.1)	39 (92.9)	0.65	7 (16.7)	35 (83.3)	0.47
	S (*n* = 23)	1 (4.3)	22 (95.7)		2 (8.7)	21 (91.3)		1 (4.3)	22 (95.7)		2 (8.7)	21 (91.3)	
Ciprofloxacin	R (*n* = 25)	1 (4)	24 (96)	0.85	1 (4)	24 (96)	0.56	2 (8)	23 (92)	0.62	7 (28)	18 (72)	**0.02**
	S (*n* = 40)	2 (5)	38 (95)		3 (7.5)	37 (92.5)		2 (5)	38 (95)		2 (5)	38 (95)	
Ofloxacin	R (*n* = 32)	1 (3.1)	31 (96.9)	0.57	1 (3.1)	31 (96.9)	0.31	2 (6.3)	30 (93.7)	0.97	7 (21.9)	25 (78.1)	0.06
	S (*n* = 33)	2 (6.1)	31 (93.9)		3 (9.1)	30 (90.9)		2 (6.1)	31 (93.9)		2 (6.1)	31 (93.9)	
Norfloxacin	R (*n* = 23)	0 (0)	23 (100)	0.18	1 (4.3)	22 (95.7)	0.65	2 (8.7)	21 (91.3)	0.52	7 (30.4)	16 (69.6)	**0.007**
	S (*n* = 4)	3 (7.1)	39 (92.9)		3 (7.1)	39 (92.9)		2 (4.8)	40 (95.2		2 (4.8)	40 (95.2)	

Abbreviations: R: resistant; S: susceptible.

## Data Availability

The data that support the findings of this study are available from the corresponding author upon reasonable request.
